# A rig for in vitro testing of the lumbar spine and pelvis simulating posterior, anterior and oblique trunk muscles

**DOI:** 10.1038/s41598-025-93599-w

**Published:** 2025-03-18

**Authors:** Georg Matziolis, Leah Bergner, Harun Hawi, Leandra Bauer, Matthias Woiczinski, Patrick Strube, Sophia Vogt

**Affiliations:** 1 Orthopaedic Department, University Hospital Jena, Friedrich-Schiller-University Jena, Campus Eisenberg, Klosterlausnitzer Straße 81, 07607 Eisenberg, Germany; 2 Experimental Orthopaedics, University Hospital Jena, Friedrich-Schiller-University Jena, Campus Eisenberg, Eisenberg, Germany

**Keywords:** Lumbar spine, Rig, Biomechanics, Trunk muscles, Bone, Muscle, Skeleton, Experimental models of disease

## Abstract

Numerous research questions require in vitro testing on lumbar spine and pelvis specimens. The majority of test setups apply forces and torques via the uppermost vertebral body with the lowermost vertebral body fixed and have been validated for kinematics and intradiscal pressure. Models without simulation of muscle traction may produce valid data only for testing conditions for which they have been validated. In vitro test setups with simulation of muscle traction would appear to be useful for conditions beyond such conditions. The aim of the present study was to describe and validate a test rig for the lumbar spine that applies the forces directly to the vertebral bodies via artificial muscle attachments and thus includes the stabilising effects of the muscles known from the literature. The artificial muscle attachments were chosen to get a stable fixation of the pulleys on the cadaver. The location of force application was as close as possible to the physiological footprint of the muscle on the bone. Three paired muscles were combined by individual linear actuators and simulated under force control (posterior, anterior and oblique trunk muscles). An optical 3D motion capture system (GOM, Zeiss, Germany) was used to measure the reposition of the entire lumbar spine and the sacrum against the ilium. At the same time, the force applied to all simulated muscles was recorded. All muscle attachments could be loaded up to a maximum force of 1 kN without failure. The following reposition of the lumbar spine could be generated by the simulated muscle traction keeping the force below each muscle’s individual strength: extension 18°, flexion 27°, lateral bending 33°, axial rotation 11°. The effects on lumbar spine reposition of the individual trunk muscles differed depending on the direction of movement. The anterior trunk muscles were the most acting for flexion/extension, at 0.16 ± 0.06°/N, while the oblique trunk muscles were the most acting for lateral bending (0.17 ± 0.16°/N) and axial rotation (0.10 ± 0.14°/N). The maximum nutation of the sacroiliac joint (SIJ) was on average 1,2° ± 0,2°. The artificial muscle attachments to the vertebral bodies proved to be withstand physiologically occurring forces. The range of motion generated in the test rig was physiological. The SIJ nutation determined and the direction of action of the muscle groups correspond to literature data. The order of the individual muscle effects on lumbar spine reposition corresponds to the distance between the muscle insertions and the physiological centre of rotation. In conclusion, taking into account the limitations, the lumbar spine test rig presented here allows the analysis of movements of the lumbar spine and pelvis resulting directly from simulated muscle tractions and thus enables a test environment close to in vivo conditions.

## Introduction

Biomechanical in vitro tests using cadaveric specimens are essential, despite the advancements in numerical models, which always require validation due to the complex technical conditions present in humans, such as degrees of freedom (DOF) and material models. Spine implants in particular (screw-rod systems, cages, prostheses) must be examined with regard to stability and impact on the instrumented and adjacent mobile segments.

However, no standardized test setup for the lumbar spine has emerged in the literature. Instead, each research group develops its own setup tailored to specific research questions regarding DOF, applied forces, and measurements (force, pressure, movement). Most published setups apply axial loads to the lumbar spine through one vertebral body while fixing another vertebral body or the pelvis^1–11^. This loading can be controlled in terms of force, torque, or path, leading to passive repositioning of the lumbar spine without the influence of in vivo muscle forces. The resulting repositioning of the vertebral body and intradiscal pressures have been validated through in vivo measurements^12–14^. However, the limitations of simple in vitro setups become apparent in borderline areas or with unvalidated extrapolations in a discrepancy between predictions and in silico models. This suggests that more complex in vitro test setups that simulate muscle traction would be beneficial^15^.

An example of this is the motion of the sacroiliac joint (SIJ), which has only been measured indirectly in vivo and is therefore subject to errors. Both in vivo and in silico models predict maximum ranges of motion that can differ by a factor of up to 50 (0.04° to > 2°)^16^. To date, there has been no direct and accurate measurement of SIJ motion on cadaver specimens combined with a simulation of the muscle forces acting on the lumbar spine.

A recent in silico study by Hadagali et al. demonstrated that motion induced by external loads applied through traditional test rigs differs significantly from that resulting from muscle contraction in the cervical spine^17^. The kinematic response of the cervical spine and intervertebral disc forces were more closely aligned with in vivo data when spine motion was induced by muscle contraction rather than external loads. While these differences may be minimal for many scientific inquiries, they could be significant for experiments concerning the influence of muscles on the lumbar spine. Various studies have indicated that sarcopenia is an independent risk factor for proximal junctional disease following posterior lumbar fusion^18–20^.

In contrast to the lumbar spine, the principle of simulating muscle traction via actuators is widely implemented in knee joint research (knee rigs), including in our own studies^21–23^. In these setups, the knee joint is moved through traction on the tendons of the major muscle groups, effectively replicating both femorotibial and patellofemoral kinematics under conditions close to in vivo conditions.

Despite an extensive literature review (PubMed, Web of Science), only two in vitro experimental setups for the lumbar spine were identified that similarly generate forces, torques, pressures, and motion on lumbar spine specimens by applying traction on the vertebral bodies in the anatomical vicinity of the muscle insertions^24,25^. Snijders et al. simulated muscle traction from the erector spinae, multifidus, and rectus abdominis muscles on specimens ranging from L4 to the pelvis, applying forces between 50 and 100 N and measuring the resulting positions^24^. Wilke et al. simulated the muscle traction of the multifidus, rotatores, iliocostalis + longissimus, and psoas major muscles at the vertebral body on L2—sacrum specimens^25^. However, the force was only transmitted to vertebral body L4. A constant force of 80 N was applied to each muscle pair (individually and with co-contraction of all muscles) and the resulting increase in stability of the mobile segment L4/5 was measured. As is common with most in vitro models, the specimens were manipulated using a material testing machine with defined preload and torque. In a follow-up study, Wilke et al. indicated that while frequently used, simplified boundary conditions are often acceptable, it may be necessary in basic research to incorporate the active muscle groups of the lumbar spine into the test setup^15^.

The objective of the present study was to establish a biomechanical test rig for the lumbar spine that actively loads and moves cadaveric specimens by simulating the key muscles influencing the spine. The study aimed to determine the maximum load capacity of the simulated muscle attachments and to compare lumbar spine and SIJ motion with existing literature data.

## Methods

All experimental protocols were approved by the ethics committee of the Friedrich-Schiller University Jena (number 2020–1962-Material). Informed consent was obtained from all subjects while they were alive. The authors confirm that all experiments were performed in accordance with relevant guidelines and regulations.

Direct attachment of tendons, as seen in knee rigs, is not feasible with established techniques such as metallic finger traps due to the short tendinous portions of the muscles acting on the lumbar spine^26^. As an alternative, artificial attachments of the muscles were therefore used by instrumentation of the vertebral bodies and acetabular cups with constructs fixed to them.

The localisation of the muscles in relation to vertebral body and pelvis landmarks was measured from anatomy atlases, patient CTs and in accordance with Remus et al.^27^. The artificial attachments were selected to maximise mechanical tear strength and to ensure that the location of force application was as close as possible to anatomical conditions (Table 1). The aim was to minimise the changes in length of the acting levers.

**Table 1:** Comparison of the anatomically correct and artificial origins and attachments of the simulated musculature.

Main acting direction	Trunk muscle	Anatomically correct attachment	Simulated attachment
Posterior	Multifidus	Spinous process	Head of pedicle screw between spinous and costal process
	Longissimus dorsi	Costal process	
Straight anterior	Rectus abdominis	Symphysis to costal arch	5 mm anterior of the symphysis to 150 mm anterior of T12 vertebra
Oblique	Psoas major	Anterior/lateral aspect of the vertebral body to lesser trochanter	Anterior/lateral of the vertebral body to lesser trochanter
	Quadratus lumborum	Costal process to iliac crest	Not simulated

**Fig. 1:**
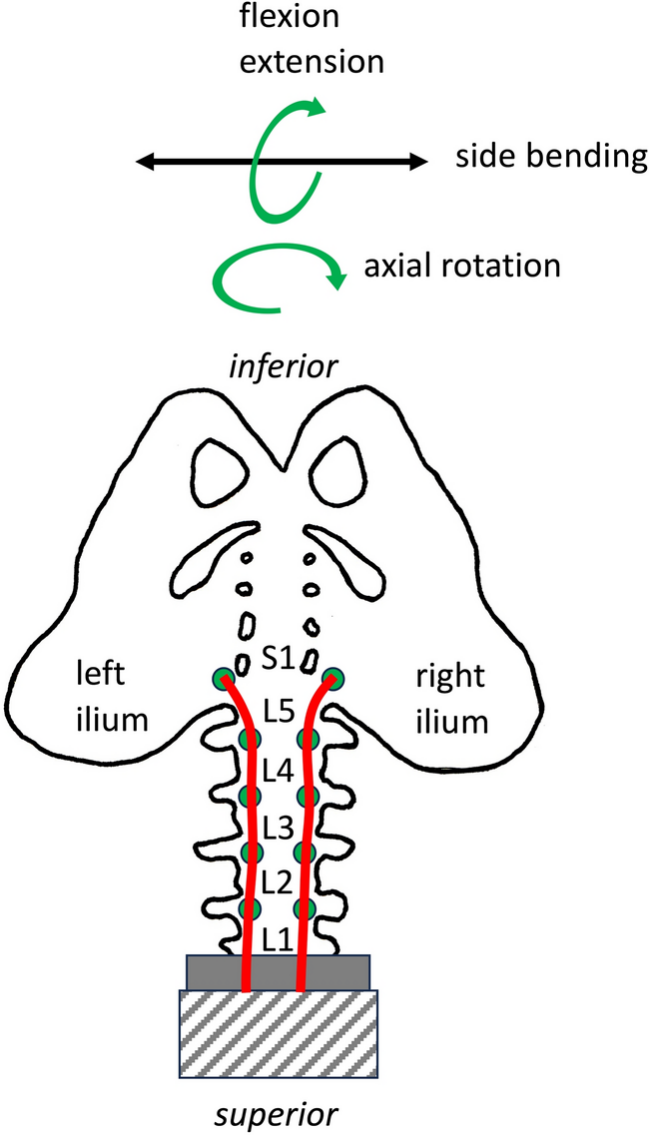
Reversed setup of the pelvis and lumbar spine with the anatomical inferior part being superior and vice versa. T12 was moulded in resin and fixed to a solid base.

An upside-down setup was chosen so that T12 was moulded in resin and fixed to a solid base (Fig. 1). This configuration allowed the entire lumbar spine and pelvis to be manipulated in space through simulated muscle traction.

**Fig. 2:**
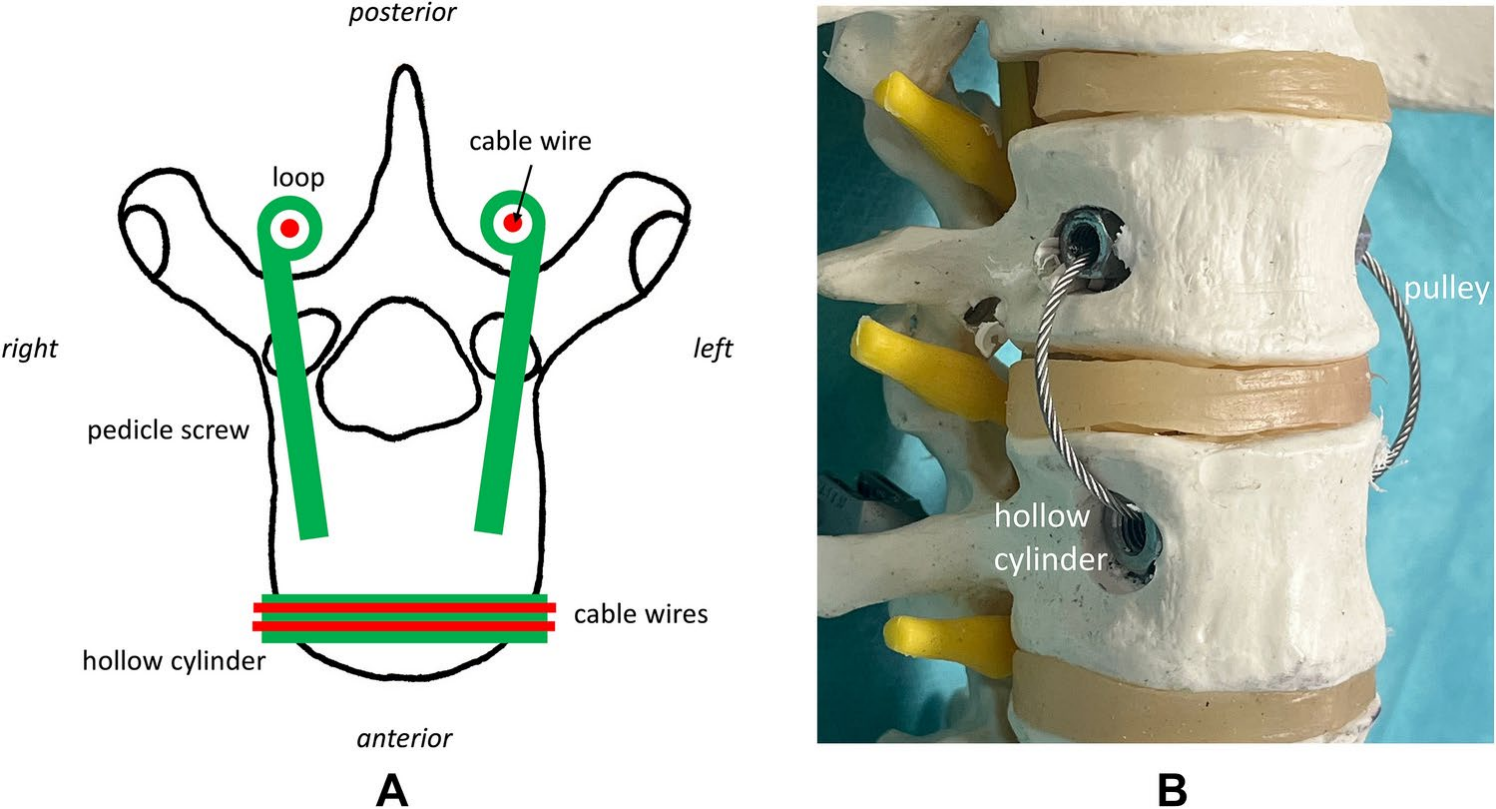
The anterior trunk actors were attached through a hollow cylinder in the anterior part of the vertebra, the posterior trunk actors were attached through eyelets at pedicle screws [(**A**) draft, (**B**) photograph with sawbone].

Artificial attachments for the anterior, posterior and oblique trunk muscles were prepared on the vertebral bodies (Fig. 2).

**Fig. 3:**
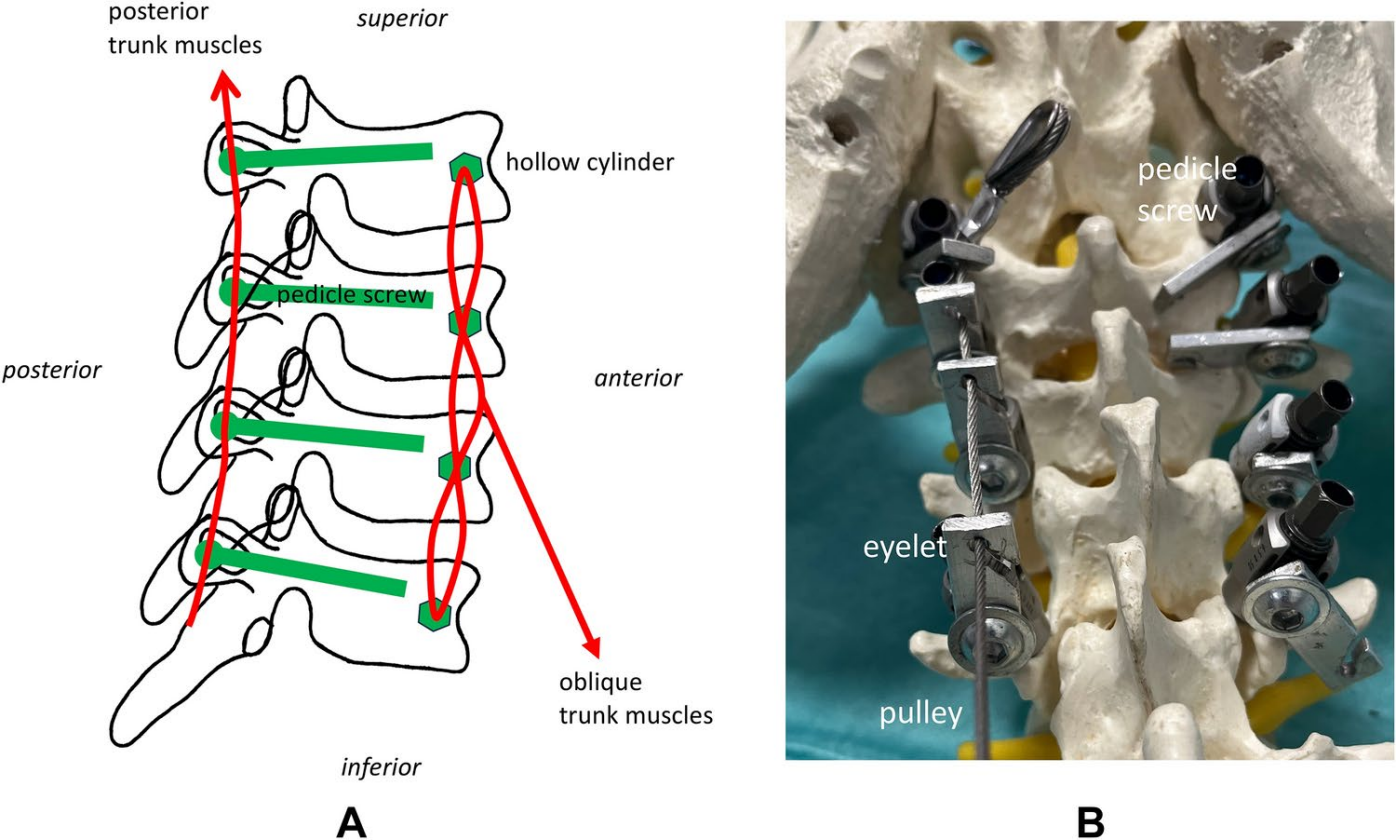
The aponeurosis of the oblique trunk muscles at the anterior vertebrae was simulated by passing a through all vertebral bodies in eight tours. A flexible wire was attached to the centre of this construction [(**A**) draft]. The pulley simulating the posterior trunk actors was placed through eylets that were fixated to pedicle screws [(**B**) photograph with sawbone].

For the posterior trunk muscles, pedicle screws (Medtronic, Memphis, USA) were inserted from the posterior in the typical manner and a flexible braided wire was passed laterally through eyelets and attached to a pedicle screw at the level of vertebral body S1 (Fig. 3). Each side had one wire guided through ball-bearing pulleys (Sprenger, Iserlohn, Germany) leading to a separate linear drive.

**Fig. 4:**
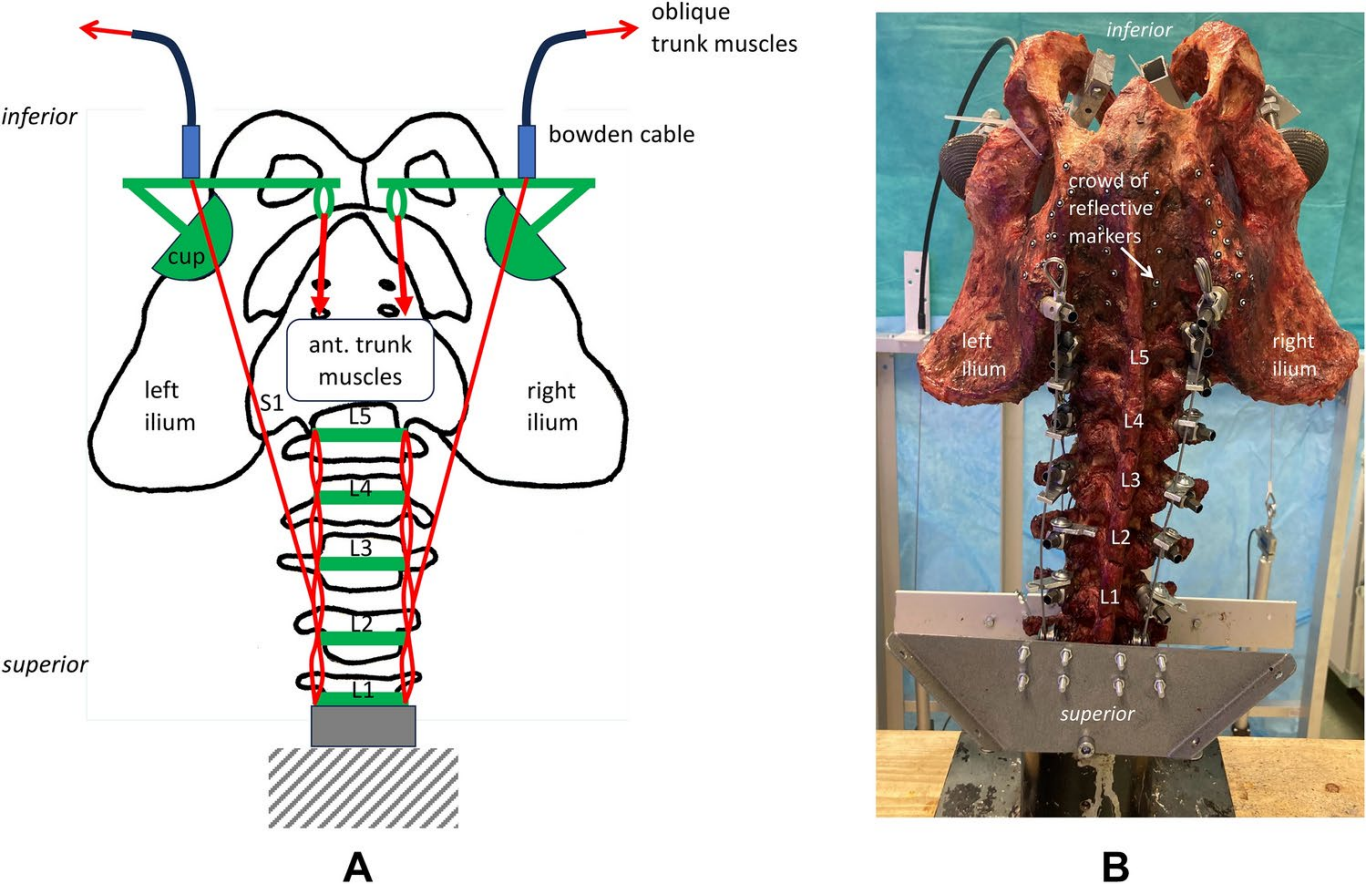
Anterior view of the setup [(**A**) draft, (**B**) photograph]. The femoral attachment of the m. psoas major was simulated by the sleeve of the Bowden cable that was fixed to the right and left acetabulum at the level of the lesser trochanter.

For the oblique trunk muscles, a press-fit sleeve was inserted into the most anterior aspect of the vertebral body (5 mm drilled, 6 mm outer diameter of the sleeve). This was done to prevent the braided wire from cutting through the vertebral body. In order to simulate the flat anterior aponeurosis of the psoas major muscle, the wire was passed through all vertebral bodies in eight loops (Fig. 3). A flexible wire was attached centrally on both sides using hooks, which connected to a Bowden cable. The use of a flexible Bowden cable was necessary because the insertion of the psoas major muscle moves with the pelvis. The sleeve of the Bowden cable was fixed to the right and left acetabulum at the level of the lesser trochanter (Fig. 4).

**Fig. 5:**
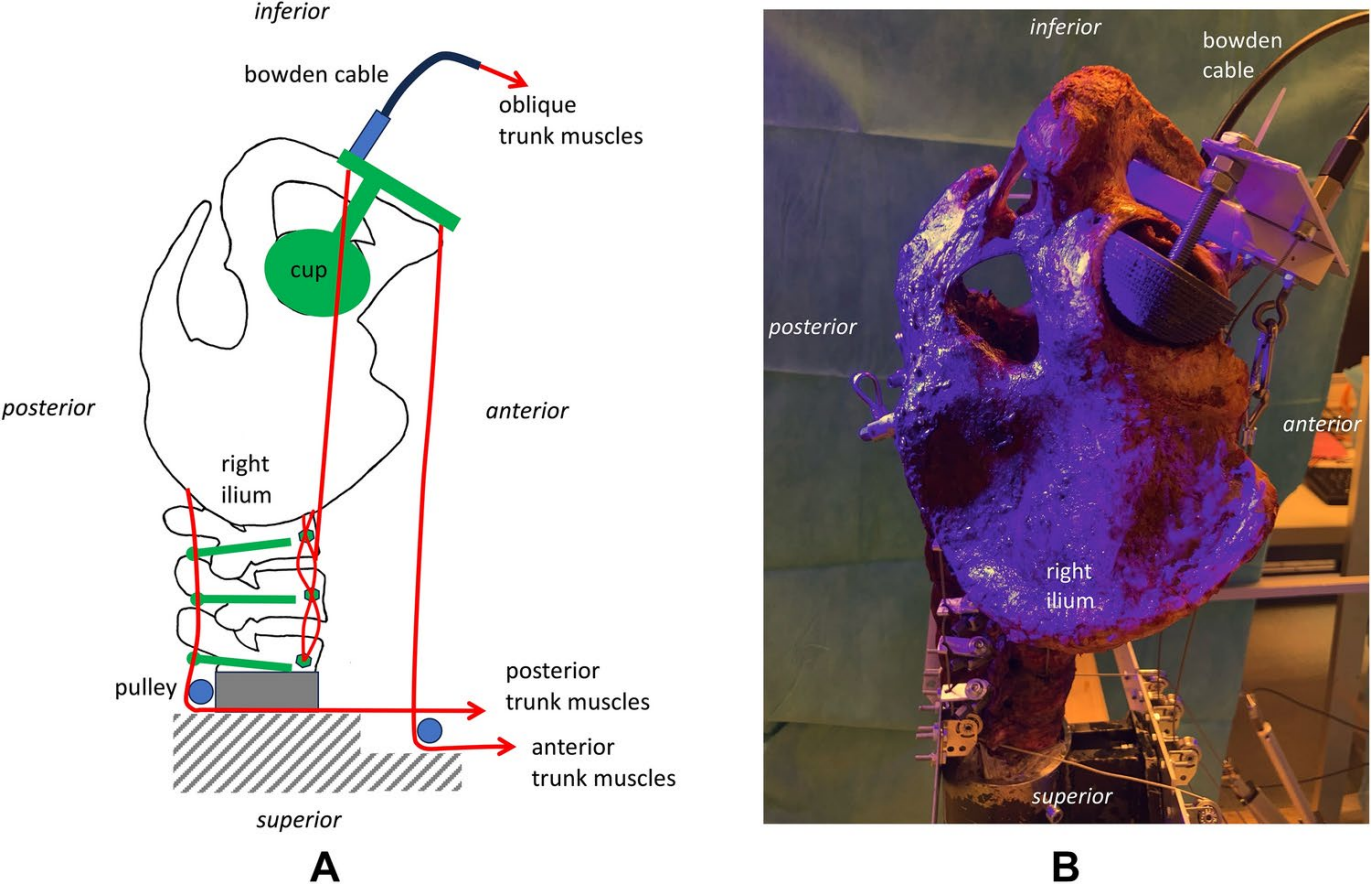
Side view of the setup [(**A**) draft, (**B**) photograph]. The anterior trunk muscles were simulated by a flexible wire cable, which was guided via pulleys at the level of the costal arch. In the pelvic region, the wire cables were attached at the level of the pubic bone.

The anterior trunk muscles were simulated by a flexible wire cable on the right and left, which was guided to the linear actuators via pulleys at the level of the costal arch. In the pelvic region, the wire cables were attached at the level of the pubic bone on the right and left via eyelets, which were connected to the right and left acetabulum via a rigid metal construction (Fig. 5).

All applied forces were measured via sensors (KD40s 1kN, ME-Meßsysteme, Germany) fixed to the linear actuators, allowing the rig to operate in both a force-controlled and path-controlled manner. To replicate in vivo conditions, all tests were conducted in a force-controlled manner. Six cadaveric specimens from T12 to pelvis were measured with the spine rig. Skin, subcutaneous tissue and muscles were removed from the specimens and care was taken to preserve the capsular/ligamentous structures of the spine, SIJ and pelvis.

For the main test, the posterior trunk muscles were simulated with up to 600 N in accordance with the physiological maximum forces, the anterior trunk muscles with up to 350 N and the oblique trunk muscles with max. 200 N. Forces were applied incrementally, with antagonistic muscle groups loaded and unloaded accordingly.The physiological maximum values were derived from estimations of the maximum muscle force based on the muscle cross-section utilizing a factor of 35—50 N/cm^2^ ^28^ as well as from validated in silico simulations^12,29^.

Finally, after carrying out the measurements, the specimens were loaded until the artificial muscle attachments failed or until reaching a maximum of 1 kN, which is the limit of the linear actuators and Bowden cable.

The position of the lumbar spine and the SIJ was determined using an optical 3D motion capture system (GOM, Zeiss, Germany). For this purpose, reflective markers were attached to the sacrum, right and left ileum using 1 mm screws, which were fully embedded into the bone. After the measurements, a CT scan of the cadavers was performed and the sacrum and ileum were segmented and the screw heads identified as the position of the reflective markers.

The position of the entire lumbar spine was broken down into the directions of extension/flexion, lateral bending and axial rotation and defined as the relative movement of the sacrum in relation to T12.

**Fig. 6:**
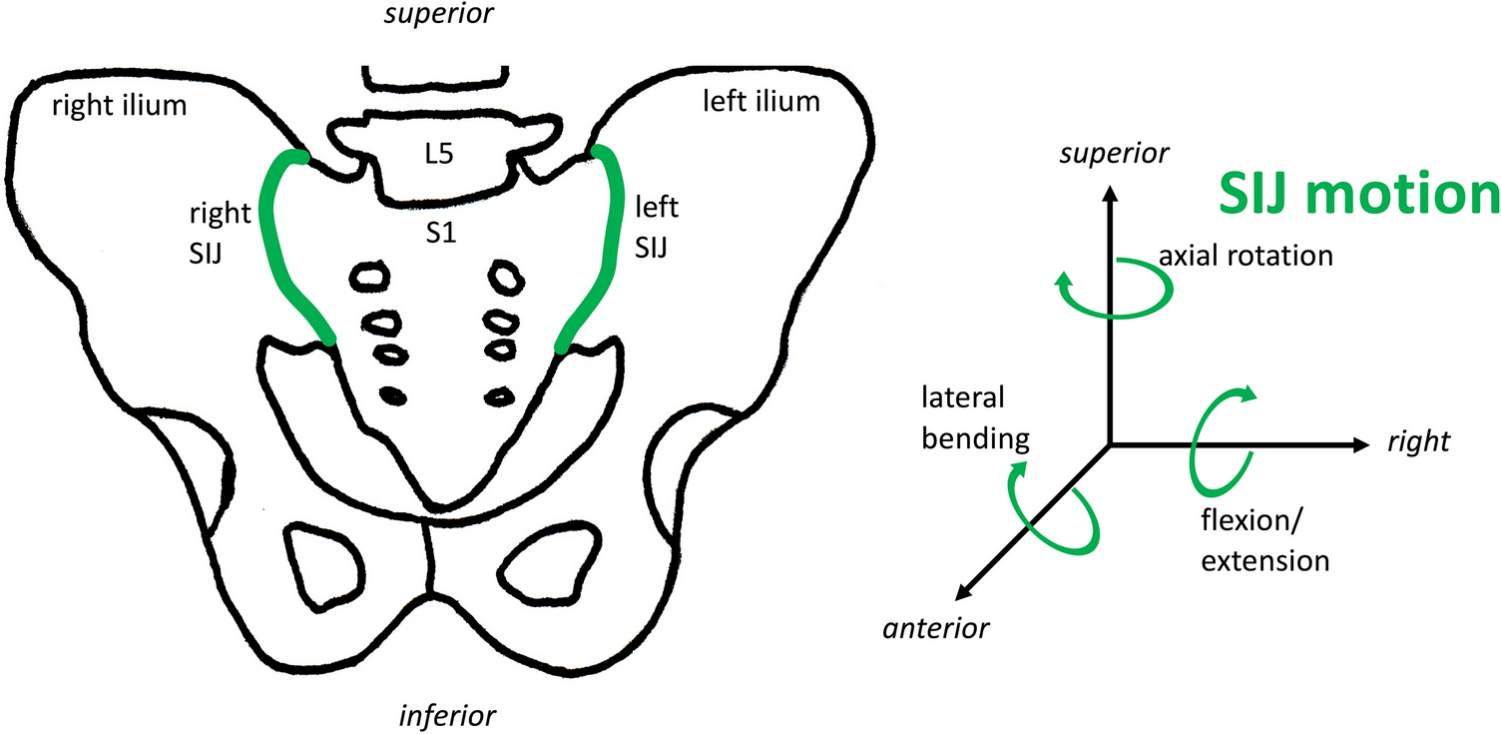
Direction of motion of the sacroiliac joint (SIJ).

The nutation of the right and left SIJ was calculated as rotation around the horizontal, vertical and transverse axes (Fig. 6).

The applied forces were recorded as analogue values by the GOM system, allowing for synchronous storage with the respective repositioning of the lumbar spine and pelvis.

### Statistics

Mean values and standard deviations were calculated for all measurements across the six tests. The effect of the individual muscles on the repositioning of the lumbar spine and pelvis was assessed using multivariate regression analysis, with muscle forces as independent variables and each measured direction of repositioning (extension/flexion, lateral bending, axial rotation) as dependent variables. The p-values determined were used to estimate the quality of correlation. The calculations were performed using custom-built Matlab script (R2022a, The MathWorks Inc., Natick, MA, USA).

## Results

All muscle attachments were successfully loaded with forces of up to 1 kN without any failure or dislodgment of the artificial muscle attachments. The simulated muscle traction was effective in producing a physiological range of motion of the lumbar spine in all spatial directions (Table 2).

**Table 2:** Range of motion of the lumbar spine in vivo^30,31^ and simulated range in the model.

	Range in vivo (°)	Simulated range (°)
Extension	33 ± 17	18 ± 11
Flexion	51 ± 12	27 ± 11
Lateral bending	62 ± 12	33 ± 7
Axial rotation	9 ± 3	11 ± 2

The contribution of individual muscles to the repositioning of the lumbar spine varied significantly across different directions. Specifically, the anterior trunk muscles exhibited the greatest influence during flexion, while the oblique trunk muscles played a more prominent role in lateral bending and rotation (Table 3).

**Table 3:** Effect of the simulated muscle force on the lumbar spine reposition as calculated by multivariate linear regression.

	Applied force range (N)	Ext/Flex (°/N)	Lateral Bending (°/N)	Axial rotation (°/N)
Posterior trunk muscles	0–600 N	0.1 ± 0.05	0.06 ± 0.04	0.02 ± 0.02
Anterior trunk muscles	0–350 N	0.16 ± 0.06	0.10 ± 0.06	0.03 ± 0.02
Oblique trunk muscles	0–200 N	0.12 ± 0.08	0.17 ± 0.16	0.10 ± 0.14

A nutation of 0.70° ± 0,13° in flexion/extension, 1.18° ± 0.24° in axial rotation and 1.11° ± 0.24° in lateral bending was determined, averaged over all cadavers.

## Discussion

The main result of the present study is that the lumbar spine test rig presented is suitable for reproducibly moving the lumbar spine via simulated muscle traction.

This study does not aim to compare the superiority or non-inferiority of this rig against established lumbar spine and pelvis rigs; rather, it serves to describe the new rig in detail and provide initial data as proof of principle.

The artificial origins and attachments of the muscles on the vertebral bodies and pelvis were sufficiently stable to transmit the forces occurring in vivo via flexible wire cables. The forces required to achieve repositioning align with the magnitudes reported in existing biomechanical models^12,32–34^.

The range of motion of the lumbar spine corresponds to in vivo conditions. However, the differences in range of motion between this rig and data from in vivo studies may be attributed to significant lumbar spine degeneration observed in all cadaveric specimens, which likely reduced flexibility and maximum range of motion^35^.

Rohlmann et al. determined the trunk muscle forces required for extension and flexion using a validated finite element model^12^. A linear relationship of approx. 0.08°/N was found between extension/flexion and the force of the erector spinae muscles. This correlation was consistent regardless of axial preload (50 N vs. 200 N), with the required force increasing exactly by the difference in preload.

Liu et al. determined a force range of 250–500 N at 60° flexion in an in silico simulation for posterior trunk muscles, resulting in a ratio of 0.12 to 0.15°/N^32^.

Senteler et al. also determined a total force of the posterior trunk muscles (iliocostalis, longissimus and multifidus muscles) of approx. 550 N at 45° flexion in silico. This corresponds to an effect of 0.08°/N^33^.

In an in silico simulation, Han et al. calculated the required total force of the posterior trunk muscles between 20° extension and 30° flexion for a mobile segment. A bi-linear relationship between muscle force and the extension/flexion of the mobile segment was found with a gradient of 0.04°/N in flexion and 0.02°/N in extension^34^. It has been shown repeatedly that the flexion of the lumbar spine is distributed evenly across the segments as a first approximation^36–38^. Therefore, the results of Han et al. can be multiplied by five for comparison with our data, as we analysed five mobile segments (T12–L5). This results in approximately 0.2°/N in flexion and 0.1°/N in extension.

In the lumbar spine test rig presented here, the effect of the extensor muscles was calculated to be 0.1 ± 0.05°/N, which is consistent with the existing literature (Table 4).

**Table 4:** Effect of posterior trunk muscles on lumbar spine flexion.

Author	Ext/flex (°/N)
Rohlmann 2006	0.08
Tao 2019	0.12–0.15
Han 2013	0.1–0.2
Senteler 2018	0.08
This study	0.1 ± 0.05

The differing effects of various trunk muscles on lumbar spine repositioning in this model are attributed to the torque generated by the tensile force in the corresponding direction, which depends on the length of the acting lever and the distance to the current center of rotation (COR). In the lumbar spine, this is located in the posterior region of the vertebral body approx. 5–10 mm anterior to the facet joints at the level of the intervertebral disc^39^. The COR is therefore closest to the insertion of the posterior trunk muscle, followed by the oblique trunk muscle and has the greatest distance to the anterior trunk muscle. The measured effects on lumbar spine repositioning align with this order (Table 3), suggesting that the COR was in the physiological position in the examined specimens.

In an in vitro study with passive loading of the sacrum against the pelvis, Hammer et al. determined the nutation of the SIJ to be 0.16° and compiled a literature review of published data^16^. These data varies considerably over more than a power of ten between 0.04°–0.1°^40,41^, 0.1°–1°^42–45^ and 1°–2°^46–48^. The variability in these values was independent of the method employed (in vivo, in vitro, in silico).

The SIJ nutation values determined in the present study, ranging from 0.1° to 0.6°, fall within the average range of published data.

The lumbar spine test rig presented here has numerous limitations. Only three paired muscle groups are simulated and these are cumulated in a simplified manner.

The model does not include the quadratus lumborum, iliocostal muscles, or oblique abdominal muscles. Although extending the model with additional simulated muscles is feasible, this is limited by the number of artificial muscle attachments that can be securely fixed to the vertebral bodies. Depending on the specific research question, it may be possible to reinforce the vertebral bodies with bone cement, allowing for more secure attachment of various muscle origins at anatomically correct positions.

Another limitation of the study is that the muscle forces were not adjusted according to the characteristics of the cadaveric specimens. Additionally, only an unphysiologically low axial preload was applied, due to the weight of the pelvis and lumbar spine. This source of error could be reduced in subsequent tests by fixing passive weights to the pelvis, but will never disappear, due to the upside-down design of the rig. Literature has reported preloads ranging from 0 to 500 N^49^. The preload used in the current test rig (approx. 50 N) aligns with studies by Panjabi et al. and Patwardhan et al.^37,50^. In the tests, the same forces were applied to the muscles of all cadavers, although it must be assumed that there are inter-individual differences, depending on the age, size, weight and constitution of the body donors.

In conclusion, despite these limitations, the lumbar spine test rig presented here enables the analysis of lumbar spine and pelvis repositioning resulting directly from simulated muscle traction.

This rig is more complicated than established and validated test setups with external load transfer to the lumbar spine. Hadagali et al. demonstrated that simulating muscle load in cervical spine studies yields kinematics and intervertebral disc forces that more closely match in vivo data compared to setups relying on external load transfer^17^.

In his PhD thesis Hadagali has shown significant differences between in vitro and in vivo determined cervical spine movement^51^. These differences were attributed to the effects of muscles and other pharyngeal tissues that act as a boundary condition for the jaw and the upper cervical spine. It is important to note that differences in force application and soft tissue boundary conditions may also lead to variations in lumbar spine movements in vitro versus in vivo. In conclusion future direction of this research would be investigating the intervertebral motions in the lumbar spine as well.

The rig has the potential to simulate altered muscle activity (e.g., in cases of sarcopenia or muscular dystrophy), which may enhance understanding of the mechanisms behind proximal junctional disease associated with sarcopenia^18–20^. A lumbar spine rig simulating posterior, anterior and oblique trunk muscles may simulate in vivo conditions more holistically than traditional rigs with passive force application.

## Data Availability

The data that support the findings of this study are available from the corresponding author upon reasonable request.
